# Probing the solar corona with very long baseline interferometry

**DOI:** 10.1038/ncomms5166

**Published:** 2014-06-20

**Authors:** B. Soja, R. Heinkelmann, H. Schuh

**Affiliations:** 1 GFZ German Research Centre for Geosciences, Telegrafenberg, D-14473 Potsdam, Germany

## Abstract

Understanding and monitoring the solar corona and solar wind is important for many applications like telecommunications or geomagnetic studies. Coronal electron density models have been derived by various techniques over the last 45 years, principally by analysing the effect of the corona on spacecraft tracking. Here we show that recent observational data from very long baseline interferometry (VLBI), a radio technique crucial for astrophysics and geodesy, could be used to develop electron density models of the Sun’s corona. The VLBI results agree well with previous models from spacecraft measurements. They also show that the simple spherical electron density model is violated by regional density variations and that on average the electron density in active regions is about three times that of low-density regions. Unlike spacecraft tracking, a VLBI campaign would be possible on a regular basis and would provide highly resolved spatial–temporal samplings over a complete solar cycle.

## Abstract (web summary)

Very long baseline interferometry is an astronomical technique that uses radio telescopes on Earth to observe extragalactic radio sources. Here, the authors show that it can be used to measure the electron density of the Sun’s corona and compare their findings to models from spacecraft tracking data.

## Graphical abstract

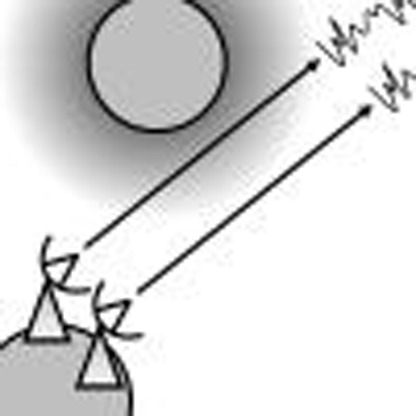

For electromagnetic waves, the Sun’s corona is a dispersive medium^1^. This results in the deflection of ray paths and correspondingly in slower group velocities^2^,^3^. The dispersive contribution to the group delay is proportional to the total electron content (TEC) along the ray path^4^. Thus, by conducting two-frequency radio observations through the Sun’s corona, the TEC can be derived and electron density models can be developed^5^. In its simplest form, such a model is radial-symmetric and usually follows a power law depending on the distance *r* (in solar radii) from the heliocenter^6^: *N*_e_(*r*)=*N*_0_ × *r*^−*β*^. *N*_0_ is the fictitious electron density at the surface of the Sun and *β* is the roughly quadratic falloff exponent. For heliocentric distances of less than four solar radii, additional terms of higher order are necessary^7^. Some models have included a heliographic latitude-dependent factor to account for the equatorial structure of the corona in times of low solar activity^2^,^8^. For the data used in this work, the single-term model is sufficient.

In the past, several techniques^9^,^10^ have been used to assess the electron density of the solar corona, for example, polarized brightness inversions from white-light coronagraph data^11^,^12^, pulsar time delay measurements^13^, emission lines observations^14^, radio interferometry on short baselines^15^ or spacecraft tracking measurements during superior conjunctions^16^.

A different technique, also capable of observing targets (in this case cosmic radio sources) with small heliocentric elongation angles, is very long baseline interferometry (VLBI^4^). VLBI measures the differences in arrival times (that is, differential group delays, often simply referred to as ‘group delays’) of signals emitted by these radio sources at several radio telescopes belonging to a global terrestrial network^17^. The signals are recorded at two frequencies in the S and X bands (centre frequencies: 2.3 and 8.4 GHz, respectively)^18^,^19^. This allows the determination of the differential dispersive effects, predominantly caused by the Sun’s corona, the Earth’s ionosphere and receiver hardware. In contrast to absolute measurements of electron content (for example, in the case of spacecraft ranging), the differential ionospheric contributions to VLBI group delays are larger than those caused by the corona. This challenge, and the techniques we developed to overcome it, are presented in detail in the Methods section.

Spacecraft measurements allow for a very precise determination of the coronal TEC, but can only probe the corona at the line-of-sight to the spacecraft during a conjunction. These observations are well complemented by coronagraph images that provide the context but are more difficult to calibrate. Compared with spacecraft tracking, VLBI has the advantage of a larger number of potential targets, which in principle enables a continuous monitoring of the corona. This is illustrated in Fig. 1a,b, which shows the apparent annual motion of the Sun with respect to the International Celestial Reference Frame 2 (ICRF2)^20^ radio sources. If only defining ICRF2 sources (that is, radio sources of highest astrometric quality) are considered, the average minimum solar elongation would be 5°. Scheduling also other ICRF2 radio sources, this angle would be decreased to 1.5° (Fig. 1c). Furthermore, usually several natural radio sources have lines-of-sight in the vicinity of the Sun at the same time, whereas opportunities to simultaneously probe the corona by observing two or more spacecraft are rare.

In this work we show that VLBI data can be used to create electron density models of the solar corona. Comparisons to models obtained by spacecraft tracking are conducted and show promising results. By analysing the geometry of the observations with respect to coronagraph images, the sensitivity of VLBI to non-spherical variations in electron density becomes evident. Finally, the potential of VLBI in monitoring the solar corona is discussed.

## Results

### VLBI data acquisition

Observations close to the Sun are necessary to study the effects of the corona. However, such observations are often of lower quality because of signal perturbations due to the corona^2^. The risk of losing observations increases for lines-of-sight close to the Sun. Also, for elongation angles less than one degree, the receiver hardware of the telescopes may get even damaged. These considerations have led to the introduction of a rather conservative cutoff elongation angle of roughly 15° in mid of 2002 (compared with 5° before 2002; ref. 21) for the International VLBI Service for Geodesy and Astrometry (IVS^18^) observing schedules.

Besides studying the corona, observations relatively close to the Sun are important for relativistic investigations^22^, in particular for the estimation of the parameter γ (from the parameterized post-Newtonian formalism) and the investigation of higher order relativistic effects. Lambert and Poncin-Lafitte^23^ presented reasons for the re-introduction of such observations into VLBI schedules, which would lead to a more precise determination of γ. The IVS decided in 2011 to dedicate 12 VLBI R&D (research and development) sessions to foster relativistic and Sun-related investigations.

The 12 experiments took place between November 2011 and December 2012. Each lasted for 24 h and featured a global network of up to nine VLBI radio telescopes (Table 1). The baseline lengths were between 920 km (Onsala, Sweden–Wettzell, Germany) and 12,400 km (Tsukuba, Japan–Concepción, Chile). The scheduling of observations followed the standard procedure for IVS VLBI sessions, but additionally included observations closer than 15° solar elongation. To avoid correlations between the parameters of the Sun’s corona, the Earth’s ionosphere and the instrumental delays in the least-squares adjustment, also observations further away from the Sun were scheduled and considered in the analysis. This way, the correlations between the coronal electron density parameter *N*_0_ and the other parameters estimated in the adjustment were <5% for all VLBI experiments.

The radio sources with ray paths closest to the Sun were preferentially observed because these observations are more sensitive to the effects of the coronal plasma. Another feature to be considered when scheduling observations is the astrometric quality of the sources, for example, indicated by their position error in the ICRF2, by their flux density and by their structure index^24^. Observations were preferentially scheduled to radio sources of higher quality (low position error, high flux density and compact structure, that is, small structure index). The duration of each individual observation (‘coherent integration time’) was chosen in a way that a certain signal-to-noise ratio (20 for X band, 15 for S band) was achieved and thus was different for every observation, depending on the baseline and radio source. On average, the integration time (scan length) for the R&D sessions was ~5 min.

**Figure 2:**
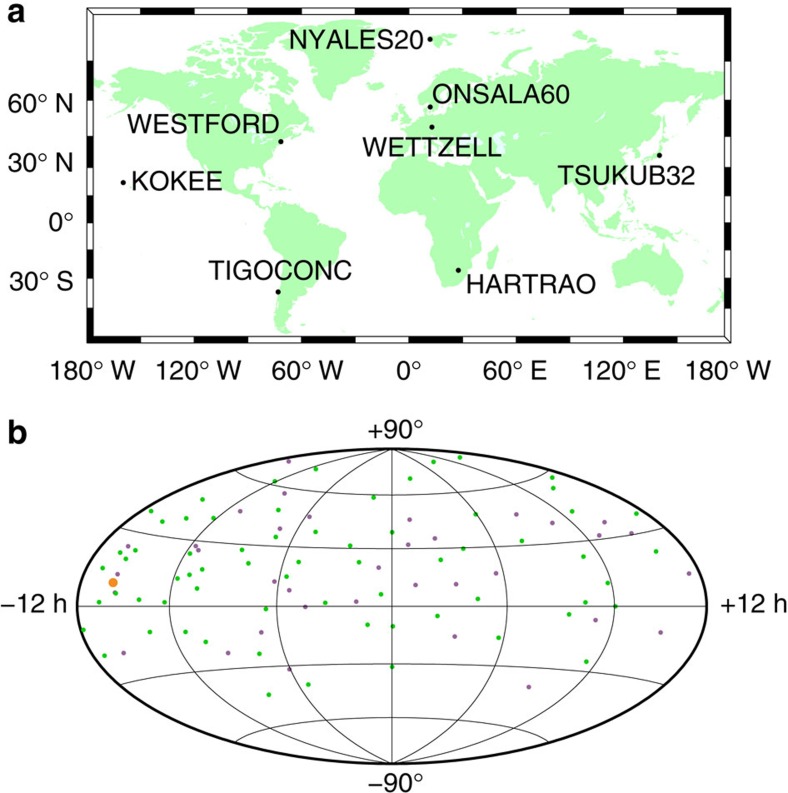
Ground and space segments for VLBI experiment RD1206. For session RD1206, plot **a** shows the VLBI station network. The radio telescopes are labelled with their IVS names. Plot **b** displays the successfully observed radio sources during this session. The orange dot represents the position of the Sun at the start of the session (28 August 2012, 17:30 UT), green dots indicate ICRF2 defining sources, purple dots ICRF2 other sources. In total, 1,558 successful observations to 106 radio sources were recorded during this session. Out of those, 193 observations were dedicated to the eight radio sources within 15° of the Sun.

During the R&D sessions, between four (RD1106, RD1107, RD1201, RD1210) and eight radio sources (RD1206) closer than 15° were successfully observed during the 24-h sessions (Table 1). The number of observations within 15° varies significantly between the sessions, depending on the pursued strategies and the numbers of available radio telescopes (cf. Tables 1 and 2). The first five sessions did not focus as much on close observations compared with the later ones. Starting with session RD1204, scans of the radio sources closest to the Sun were scheduled on a regular interval. The low number of observations during session RD1204 was due to two radio telescopes being unavailable (Westford and Kokee Park) and the radio telescope in Tsukuba having to pause because of a passing typhoon. As an example of a typical VLBI network used in the R&D sessions as well as of the corresponding space segment, Fig. 2 shows the geometry of stations and radio sources for session RD1206. The minimum heliocentric distance at which observations could be scheduled depended on the availability of high quality radio sources and is given in Table 2. For almost all sessions, observations between 4° and 6° elongation were successfully carried out. An exception is session RD1203 with no appropriate radio source closer than 10°.

The signals recorded at the radio telescopes during the R&D experiments were collected and correlated at the MIT Haystack Correlator (co-sponsored by NASA Goddard Space Flight Center). The resulting differential group delays (that is, the delays between the two stations of a baseline) for S and X band have been made available through the IVS. More information on the observables is given in the Methods section.

### Electron density models from VLBI

Using the data described in the previous section and the procedures outlined in the Methods section, we derived an electron density model for each VLBI R&D experiment. Table 3 shows the estimates and uncertainties of the power-law parameter *N*_0_. A weighted mean of the individual electron density models was computed as *N*_0_=(0.57±0.18) 10^12^ m^−3^, utilizing the inverse variances as weights. When assuming normal distribution, the *t*-test shows that this average *N*_0_ value is different from zero with a probability larger than 99.9%.

As mentioned, the closest radio sources that were successfully observed in these 12 sessions had heliocentric elongation angles just below 4°. With no closer observations available, the parameters *N*_0_ and *β* are significantly correlated. To avoid singularities, *β* was kept fixed in the estimation procedure on different values between 2 and 2.6 (typical values from previous models^16^). No significant differences in the resulting electron densities and their s.e. could be seen from the heliocentric distances at which VLBI observations were made. For the values shown in Table 3, the theoretical value *β*=2 for constant solar wind velocity was chosen^6^. To estimate both power-law parameters simultaneously, closer observations would be necessary.

For increased robustness in the estimation, a simple outlier test based on the normalized residuals was applied. This way, on average 0.4% of the observations were excluded from the estimation. The total number of observations for each session is given in Table 2. All observations not detected as outliers were included in the least-squares adjustment, which allowed for precise estimates of the ionospheric parameters and the instrumental offsets. In the same adjustment, the corona parameter *N*_0_ was estimated from the observations within 15° elongation. At 15° elongation, assuming the average electron density model obtained in this paper, a very long baseline would have a differential coronal delay of ~10 ps (3 mm), which is in principle detectable by VLBI. Tests have been made using a smaller elongation threshold to create the electron density model. For a maximum elongation of 8°, for instance, the average model was computed as *N*_0_=(0.72±0.26) 10^12^ m^−3^. These results are less precise due to a significantly smaller number of usable observations: Session RD1203 does not include any observations within 8°, and a few other sessions contain only a very small number. During RD1107, for instance, source 1657–261 at ~4° elongation was only observed twice. For these sessions, no or significantly less accurate estimates can be given.

**Figure 3:**
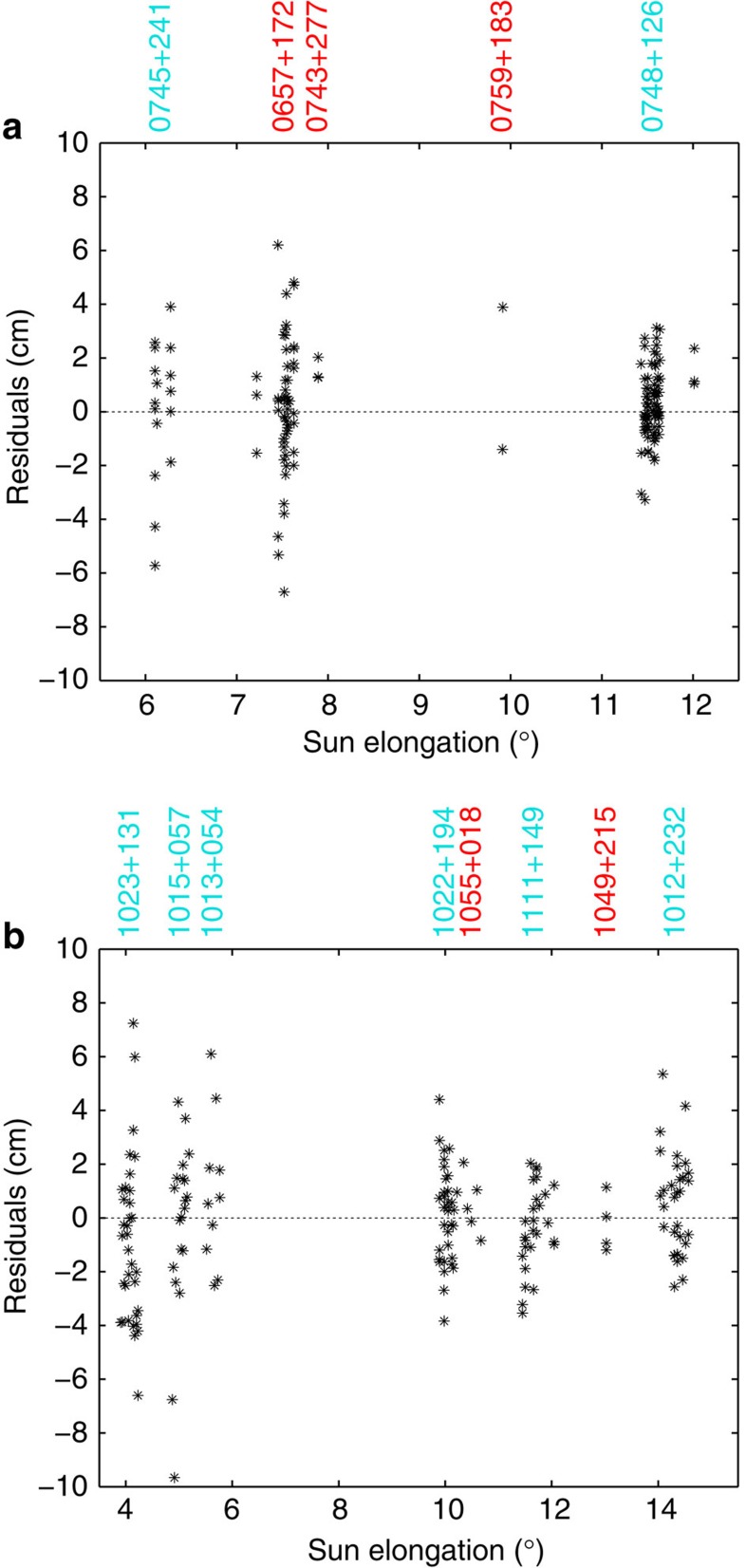
Residuals of the least-squares adjustment. For sessions RD1205 (**a**) and RD1206 (**b**), the residuals of observations within 15° elongation obtained in the least-squares adjustment are shown. The residuals (‘observed minus computed’) are plotted against the solar distance and stay within ±10 cm, corresponding to ±0.3 ns. Above each plot the names of the observed radio sources are given. The turquoise or red colours indicate that the ray paths passed through low or high density regions of the corona, respectively.

A distinct detection of the coronal contribution for each measurement was not possible in a sense of directly relating the estimated parameters to the observations. This can only be done indirectly via the residuals (observed minus computed) of the least-squares adjustment. As an example, the residuals for session RD1205 and RD1206 are plotted in Fig. 3. The residuals were on average smaller when estimating the effect of the solar corona compared with not considering it (12% improvement for the observations within 15° elongation).

To judge whether it is warranted to include the parameter *N*_0_ in the model describing the observations, the Akaike Information Criterion (AIC^25^) was computed for each session: AIC=2*k*+*χ*^2^+*C* with the number of model parameters *k*, the weighted sum of the squared errors *χ*^2^, and a constant *C*. Additionally, AIC values were derived for a least-squares adjustment in which only the ionosphere and receiver hardware parameters were estimated. The difference in AIC, given in Table 3 shows that for 11 out of 12 sessions, more information is retained when the corona parameter *N*_0_ is included. The exception is RD1207, for which the AIC is slightly in favour for excluding *N*_0_ with a relative likelihood of 65% for the model including *N*_0_ with respect to the one without it (Table 3). This difference between estimating *N*_0_ and excluding it is not very distinct compared with most other sessions: for 7 out of the 11 sessions for which the inclusion of *N*_0_ is favourable the relative likelihood is <10%. The different outcome for RD1207 is also evident in the reduced *χ*^2^ (Table 3) that indicates that for this session the worst goodness of fit was obtained. The reasons could be that the closest ray paths were only at 6.1° solar elongation and that the total number of observations was less compared with most other sessions what might have led to less precise ionospheric modelling.

Soja *et al.*^26^ performed comparisons with external ionospheric data from GPS to test the approach of estimating the parameters of the ionosphere from VLBI data. In general, a good agreement between the vertical TEC obtained from the two techniques was found, with maximum differences close to the stated accuracy of the GPS data. By using the GPS data to eliminate the ionospheric effects on the VLBI data, it was possible to estimate the electron density of the corona which could then be compared with that derived from VLBI data only. While for the individual session-based models, differences between the two approaches were evident, the average model from all 12 VLBI experiments agreed by 95%. It was concluded that the approach using only VLBI data leads to more reliable and precise results. It has to be noted that in this publication^26^, the assumption of *β*=2.3 leads to different numerical values for *N*_0_ compared to what is shown in Table 3. This assumption has a negligible effect on the electron density at the heliospheric distances at which the VLBI data were recorded.

## Discussion

Our results comprise the first solar corona electron density model successfully developed from VLBI group delays and provide a measure of the potential of VLBI for probing the solar corona. Compared with spacecraft measurements, a disadvantage of VLBI is the low signal strength of cosmic radio sources, which makes successful dispersive group delay measurements unlikely at elongation angles of <2°. For instance, observations of the radio source 1030+074 between 1.8° and 2.3° elongation, during the VLBI R&D experiment on 28 August 2012, did not provide useful data in the S band and could therefore not be included in the analysis. The VLBI sessions discussed here took place during a period of medium solar activity. However, for times of low solar activity, two-frequency group delays at just above 2° have been successfully observed^21^. One strength of VLBI is that observations to radio sources in the vicinity of the Sun are possible on a regular basis and do not depend on the coincidence of a spacecraft’s superior solar conjunction.

**Figure 4:**
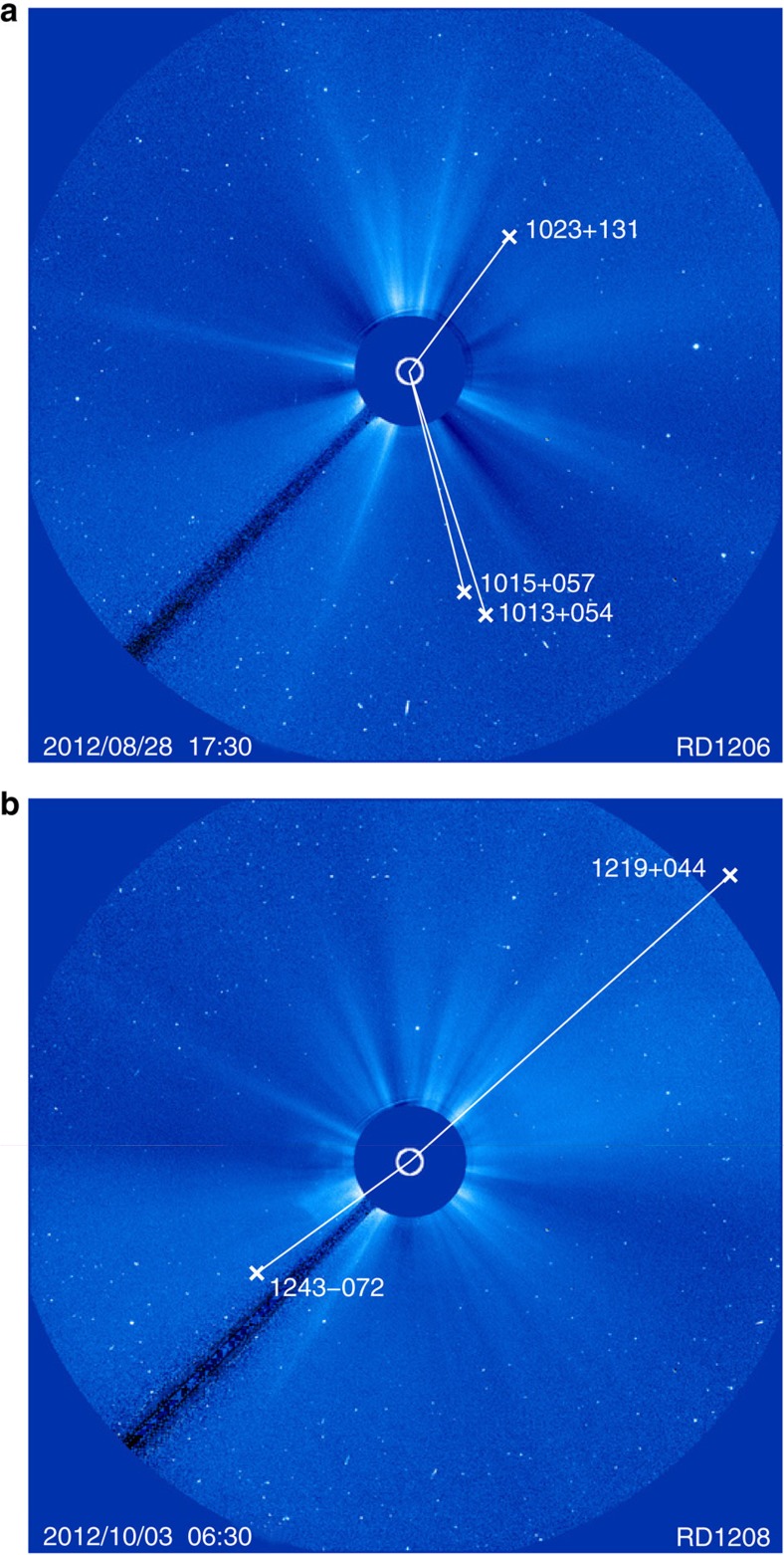
Radio source geometry and coronagraph images. For the VLBI experiments RD1206 (**a**) and RD1208 (**b**), the positions of the radio sources in the vicinity of the Sun are overplotted on LASCO C3 coronagraph images^28^. The field of view is 32 solar radii (8.7° elongation). The white circle indicates the position of the Sun limb, white crosses those of the radio sources. The dark beam on the lower left in both panels is not a feature of the corona, but the pylon holding the occultation disc of the coronagraph.

The coronal electron density distribution is expected to be correlated with the solar activity cycle^1^. However, indicators for solar activity (for example, sunspot numbers or solar flux indices) describe the integrated activity of the Sun, whereas the VLBI delays are only affected by the coronal structure in the vicinity of the ray path^7^,^8^,^27^. This is shown in Fig. 4. Here, as an example, the source positions during two VLBI experiments are compared with images from the Large Angle and Spectrometric COronagraph (LASCO^28^) C3 coronagraph onboard the Solar and Heliospheric Observatory (SOHO) spacecraft. During VLBI session RD1206, the radio sources were located in regions of low white-light intensity and we obtained a lower electron density than the mean VLBI model. For RD1208 it was the opposite: sources were found in high density (streamer) regions and the value of *N*_0_ was higher. The animated LASCO images for the duration of the VLBI observations during session RD1206 and RD1208 are provided as Supplementary Movies 1 and 2, respectively. For sessions with the radio sources spread over diverse regions, the resulting electron densities were closer to the average model (for example, RD1205). Thus, these violations of the assumption of a spherical electron density distribution explain some of the scatter found in the *N*_0_ values in Table 3.

The variations in precision of the estimated *N*_0_ values are to some extent dependent on the number of observations close to the Sun. The sessions RD1205 and RD1206 had the largest numbers of observations within 15° elongation (186 and 193, respectively) and consequently the electron density models could be derived with the highest precision (±0.1 and ±0.3 × 10^12^ m^−3^, respectively). Sessions RD1201 and RD1204 with 31 and 32 observations, respectively, obtained less precise results (±1.3 and ±1.0 × 10^12^ m^−3^). Session RD1203 shows that also the minimal observed elongation angle affects the precision: with no sources closer than 10° elongation available, the precision drops considerably (±1.8 × 10^12^ m^−3^).

At the time of some of the VLBI experiments, coronal mass ejections (CME) took place. During the last few hours of session RD1201, a CME reached the lines-of-sight to radio sources 1958−179 and 2008−159 (Supplementary Movie 3). The additional plasma from the CME affected also the estimate of *N*_0_, which is larger than for other sessions. RD1205 features a strong CME (Supplementary Movie 4), but the VLBI experiment ends before it can reach the ray paths to radio sources 0743+277 and 0745+241. During RD1208, a minor CME passes the lines-of-sight of radio source 1243−072 and the estimated electron density during this session is larger than average.

**Figure 5:**
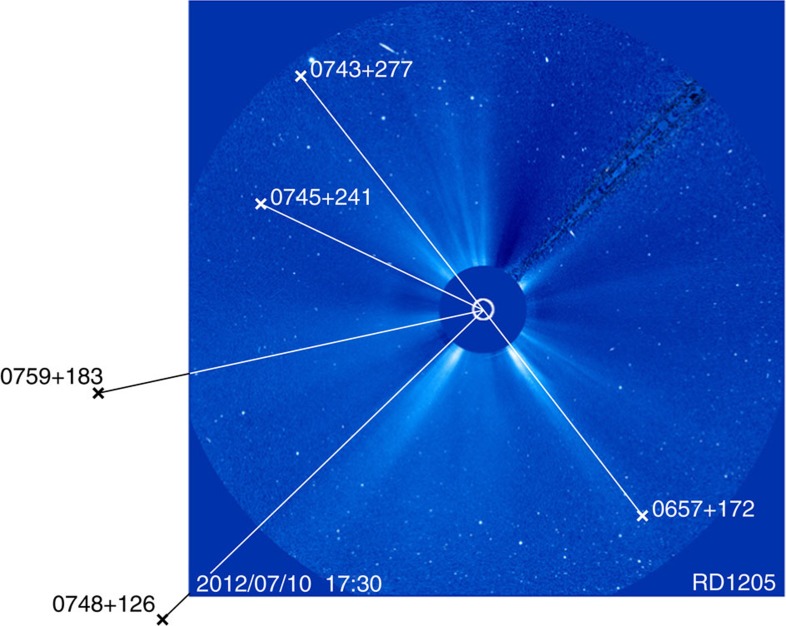
Radio sources and the corona during RD1205. Similar to Fig. 3, the geometry of radio sources with respect to the corona is shown. Here, the situation at the start of session RD1205 is displayed and also radio sources just outside of LASCO C3’s field-of-view are plotted.

The assumption of spherical symmetry limits the accuracy of the electron density models derived by VLBI. Therefore, for the two sessions with the largest number of observations close to the Sun (that is, RD1205 and RD1206) a different parameterization was tested. With the aid of the LASCO C3 images, the observations within 15° elongation were separated in two groups depending on whether the lines-of-sight pass through low or high-density regions. As the position of the Sun changes with respect to the radio sources during the 24 h duration of the sessions and the coronal electron density has temporal variations, this categorization is sometimes ambiguous. Furthermore, for the observations outside the field-of-view of the C3 coronagraph, the visual information has to be extrapolated. Figure 5 shows for the start of session RD1205 all radio sources with ray paths within 15° elongation together with the LASCO C3 image. The 116 observations of radio sources 0745+241 and 0748+126 are mostly in the low-density corona, the other 70 observations in high-density regions. The categorization of the radio sources is also indicated in Fig. 3. For each set of observations *N*_0_ was determined: (0.2±0.4) 10^12^ m^−3^ (low density) and (0.7±0.4) 10^12^ m^−3^ (high density). For comparison, the overall value for *N*_0_ for this session is (0.5±0.3) 10^12^ m^−3^ (Table 3).

During session RD1206, only 1049+215 and 1055+018 were located behind coronal plasma of higher density. These two radio sources, both at solar elongations larger than 10°, were observed 50 times. The six radio sources with ray paths in low density regions accumulated 143 observations. Three of these radio sources are seen in Fig. 4a. The resulting *N*_0_ value for the low-density regions is (0.3±0.1) 10^12^ m^−3^, which corresponds to the overall value for the session. The observations in higher density regions had a low impact on the overall value because of the larger angular distance from the Sun and the lower number of observations. Still, it was possible to derive an individual *N*_0_ value from these observations, although less precise: (1.0±0.6) 10^12^ m^−3^. Similar to session RD1205, the electron density is larger by a factor of about three.

**Figure 6:**
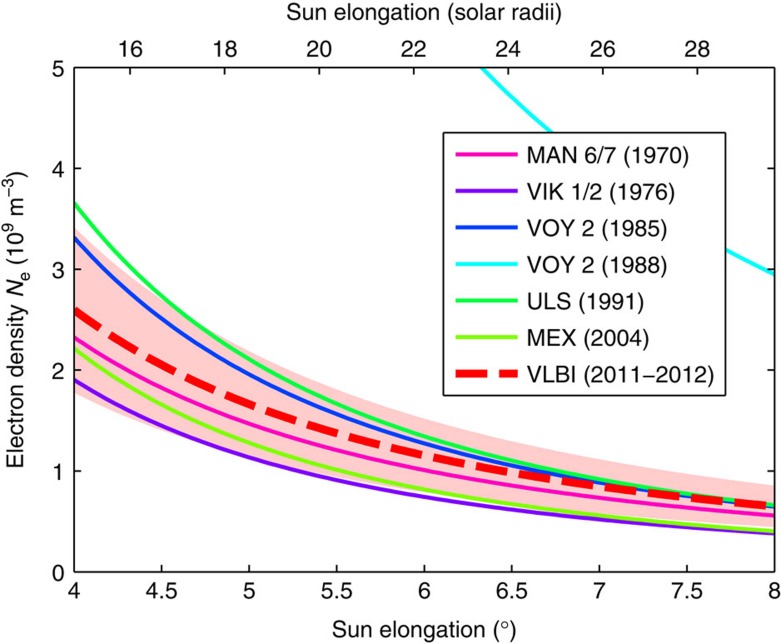
Electron density models of the solar corona. The plot shows a comparison of the electron density models from VLBI (2011/2012) and various spacecraft missions between 1970 and 2004. For the VLBI model, the 1*σ* s.e. are plotted (shaded area). The spacecraft models are taken from the study by Bird *et al.*^16^ In this reference, also models from the data of Rosetta (2006) and Mars Express (2008) are listed, which have been excluded in this plot for better clarity. They are in good agreement with the other models. Further details about some of the underlying spacecraft tracking data can be found in the study by Bird *et al.*^6^

In the case of the mean electron density model *N*_0_=(0.57±0.18) 10^12^ m^−3^, the effects of different observation geometries and transient events are, to some extent, averaged out. Figure 6 shows the comparisons of the model created from VLBI data to previous models developed from spacecraft tracking measurements during superior solar conjunctions. The models obtained from spacecraft data were determined at various periods of different solar activity. For instance, during the Ulysses conjunction in 1991, a solar maximum took place^6^. The Mars Express conjunction in 2008 happened during very low activity^27^. The models, therefore, cover a range of realistic electron densities of the Sun’s corona. The model from VLBI agrees very well with the results from the spacecraft missions, especially when considering that the VLBI data, on average, were acquired during a period of medium solar activity (between Nov 2011 and Dec 2012). The data from the 1988 conjunction with Voyager 2 (uppermost curve in Fig. 6) stands out as most ranges were recorded when the signal ray path was passing a dense coronal streamer^6^. Evidently, the electron density models from spacecraft tracking are affected by regional coronal structures to a similar extent as the individual VLBI models in Table 3.

In the future, the determination of the Sun’s corona electron densities using the VLBI technique could be optimized in a variety of ways. The next release of the ICRF^29^, probably in 2018, will include even more radio sources than the current one. Thus, the number of candidate radio sources for Sun-related investigations is very likely to increase significantly. Also, from a technological point of view, significant improvements are expected with the new-generation VLBI system VLBI2010 (refs 4, 30) and its future network of VLBI stations, the VLBI Global Observing System (VGOS). Instead of group delays, phase delays will be observed that can be used to study turbulence and solar wind properties in the corona^31^, in addition to estimating electron densities^15^.

Considering all upcoming improvements, the VLBI technique can become a powerful tool for monitoring the electron density of the Sun’s corona. A requirement, however, is the availability of observational data close to the Sun. In this respect, a major milestone was reached at the end of 2013, when the IVS decided to lower the cutoff elongation angle from 15° to 4° for standard geodetic VLBI sessions. Our results imply that VLBI will be of great benefit to future investigations of the solar corona.

## Methods

### Differential dispersive group delays observed by VLBI

The basic geodetic VLBI observable is the differential group delay *τ*=*t*_2_–*t*_1_, *t*_*i*_ denoting the arrival time of radio signals at two-radio telescopes^4^. It is obtained by cross-correlating the signals recorded at both stations^32^. Group velocities of electromagnetic waves in dispersive media are slower compared with the propagation in vacuum, causing an additional dispersive contribution *τ*_disp,*f*_ (ref. 33). This delay depends on the frequency *f* of the radio wave and thus can be assessed by observing at two frequencies at the same time^34^. For this reason, VLBI observations are routinely performed in the S band (2.2–2.4 GHz) and the X band (8.2–8.95 GHz). The corresponding frequency-dependent delays between the two stations are given by *τ*_*f*_=*t*_2,*f*_–*t*_1,*f*_, with *f* denoting either S or X band. At these frequencies, the dependence of the delay on the frequency may be assumed to be proportional to *f*^−2^ and no higher order terms have to be included^35^. By forming a linear combination of the observed group delays in the S and X bands, the dispersive contribution in the X band can be determined^36^:


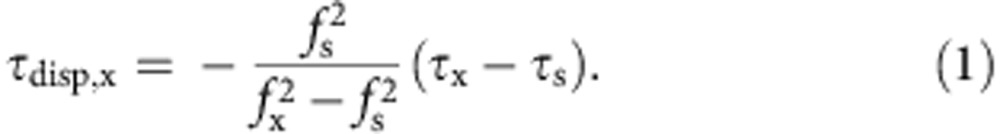


The dispersive delays are due to the intergalactic and interstellar media, as well as the Earth’s ionosphere, receiver hardware, and, in the case of observations close to the Sun, the corona. As VLBI is a differential method and the baseline lengths are shorter than the diameter of the Earth, the effects of media with small gradients in electron density are negligible. Thus, following Hobiger^36^, we assume that the effects of the intergalactic and interstellar media can be neglected. At the heliocentric distances the VLBI data were observed, the effects of path separation due to different propagation of the S and X band signals^2^ in the coronal plasma can be considered negligible. The instrumental dispersive delays arise due to different propagation of the S and X band signals in the receivers^34^.

Effects in turbulent plasmas like angular^37^ or spectral broadening^2^ can lead to less precise observations or even non-detections during the correlation of the signals. Density irregularities on longer time scales cause some of the scatter seen in the VLBI measurements (Fig. 3). They do not systematically affect the coronal contribution to the observed group delays and their effect is thus reduced by including several observations from different points of time in the parameter estimation. Effects of fluctuations with periods less than the coherent integration time are reduced significantly due to the averaging nature of the group delay observable.

Collecting the various effects, the observation equation reads:


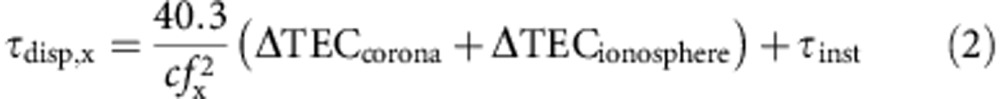


with *c* denoting the vacuum speed of light, TEC the total electron content and *τ*_inst_ the instrumental biases. The leading Δ denotes the difference between the respective values for the two radio telescopes. On the left hand side there is the observation and on the right hand side the unknown quantities. Before it is possible to solve for these unknowns by adjustment, it is necessary to parameterize them in a way to reach redundancy. The instrumental delays can be assumed to be constant over the duration of usual VLBI sessions (that is, about 24 h), except for rare cases^36^ not occurring in the data discussed in this work.

### Absolute and differential measurements of TEC

For this study, the electron density *N*_e_ in the Sun’s corona is of interest. Simplified, it can be described by a radial power law in the form of:


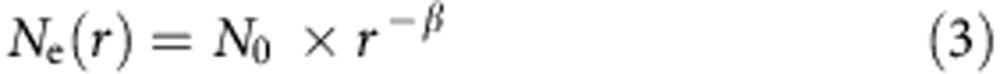


with the fictitious electron density at the surface of the Sun *N*_0_, the distance from the heliocenter *r* in solar radii (*R*) and the radial falloff parameter *β* (ref. 7). The coronal TEC included in Equation (2) can be assessed by numerically integrating the power-law model from Equation (3) along the ray path *S*:


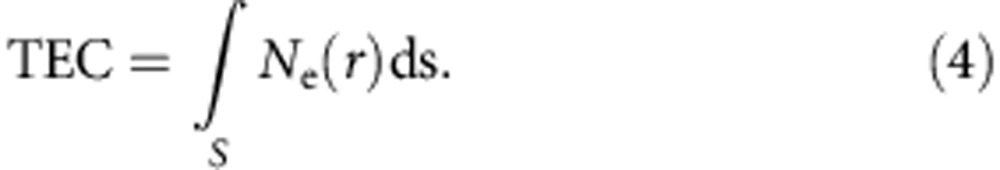


Compared with the electron density *N*_e_ that falls off approximately as *r*^−2^ (ref. 6), the TEC, directly detected by spacecraft tracking, decreases roughly as *p*^−1^ (*p* is the impact parameter, the smallest distance between the ray path and the Sun). Given that for VLBI baselines *p*_1_≈*p*_2_≈*p* holds, it can be shown that ΔTEC(*p*)=TEC(*p*)(−Δ*p*)/*p*. This means that ΔTEC, which is detected by VLBI, is proportional to ~*p*^−2^. At an elongation of 4° (*p*=15 
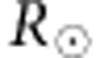
) and a projected baseline of Δ*p*=1,000 km, the differential TEC is smaller than the absolute TEC by a factor of 10^4^.

The most significant difference between using absolute (spacecraft tracking) or differential measurements (VLBI) is the handling of the ionosphere. For a single ray in zenith direction from a radio telescope close to the equator, the TEC of the ionosphere can reach up to 100 TECU (1 TECU=10^16^ electrons per m^2^). For observations at low elevations, the TEC is larger, for example at an elevation of 5° the factor is ~2.7 (ref. 38). For stations at mid or high geographic latitudes the ionospheric TEC is smaller^36^, usually not exceeding 50 TECU in zenith direction. The coronal TEC for a single ray passing the Sun at a distance of 40 
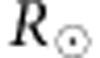
 (~11° elongation) is usually a few thousand TECU, and reaches 10^4^ TECU at 10 
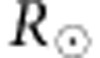
 (ref. 27). Thus, in the case of absolute TEC measurements the contribution of the Earth’s ionosphere is minor and can be neglected.

For differential observations on a global scale (that is, baselines longer than a few thousand kilometres as in the case of VLBI), the ionosphere can cause a differential delay of up to 3 ns (corresponding to a distance of ~1 m) in X band. The effect of the Sun’s corona is smaller: Assuming a global baseline, X band observations at a distance of 10 
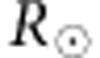
, and the electron density model from the DSMS Telecommunications Link Design Handbook^5^, the differential coronal delay is <1.5 ns. For most observations, the effect of the corona is significantly smaller.

Therefore, without knowledge about the ionospheric TEC it is not possible to create meaningful electron density models of the solar corona using VLBI data. To overcome this problem, we parameterize the ionospheric TEC in a way enabling us to estimate it together with the unknown parameters of the corona and the instrumental biases in a least-squares adjustment, as described in the next section.

### Estimating the ionospheric TEC from VLBI data

The task of estimating the ionospheric TEC from VLBI data is achieved by relating the ionospheric TEC (from here on designated as slant TEC, STEC) for each observation to the vertical TEC (VTEC) above the VLBI antennas. To convert the STEC to the station-based VTEC we follow a method first proposed by Kondo^39^ and later developed by Hobiger *et al.*^34^ The first assumption is that all charged particles of the ionosphere are compressed into a thin layer at the height of the F2 layer (under normal conditions this is the height of the largest electron density^36^). Using an ionospheric mapping function *mf*, the STEC can be converted to the VTEC at the point of intersection between the ray path and the thin layer (that is, the ionospheric pierce point, IPP), denoted by a prime:


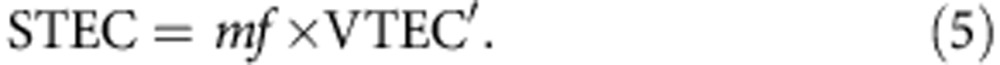


The ‘modified single-layer model mapping function’^38^ is used with the scaling factor *α*=0.9782 for zenith distances *z*, the height *H*=506.7 km of the thin-layer ionosphere and the Earth radius *R*_e_=6,371 km:


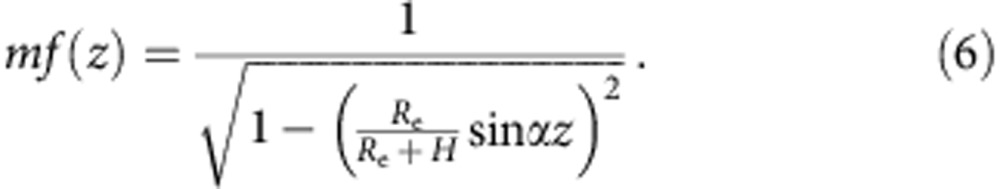


The factor *α* is introduced to approximate the mapping function of a Chapman profile (which would require numerical integration) to <1% (ref. 38). The horizontal distance between the radio telescope (geographic coordinates *λ*, *ϕ*) and the IPP (*λ′*, *ϕ′*) can be up to 2,000 km for low elevation angles (assuming zenith distances of up to 85°). To assess the VTEC at the station, further simplifications are necessary, leading to the following equation^34^:


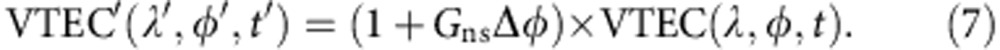


Here, the difference in latitude Δ*ϕ* is considered by introducing a linear north-south gradient *G*_ns_. Further improvement can be achieved by using two gradients, one for observations with azimuths between −90 and 90 degrees (north gradient) and one for those between 90 and 270 degrees (south gradient). We apply this two-gradient approach as recommended by Dettmering *et al.*^40^

Assuming that the ionospheric VTEC distribution co-rotates with the apparent movement of the Sun (360° per day) for durations below 1 hour, it is possible to relate the difference in longitude (unit: degrees) to a difference in time (unit: hours):


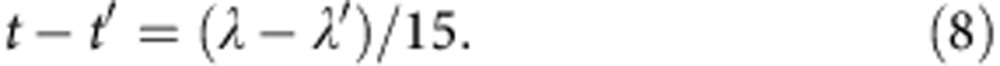


Generally, this method of estimating station-based VTEC from VLBI observations works best if the coordinates are first transformed to a geomagnetic coordinate system. The errors due to the various assumptions increase with growing zenith distances, making it necessary to down-weight low elevation observations in the least-squares adjustment^36^.

To reach redundancy in the adjustment, we parameterize the VTEC using piece-wise linear functions with adaptive interval lengths^36^. The interval borders are determined in a way that every interval contains the same amount of observations. The advantage is that during periods with many observations, the temporal resolution of the estimated VTEC is higher. For times of lower observation density, less intervals are necessary, thus reducing computation time. Compared with a parameterization with regularly spaced intervals, no constraints are necessary to prevent singularities due to unsupported parameters. While in theory, only two observations per interval would be necessary for redundancy, solutions are more stable and smooth with a larger number of observations per interval. Hobiger *et al.*^34^ recommend a minimum of eight observations per interval in order to achieve reliable estimates of the ionospheric VTEC. In our computations, we tested 10, 15 and 20 observations per interval and found that 15 observations, corresponding to a mean temporal resolution of 45 min, offered a good compromise of temporal resolution and reliability.

The stochastic model is obtained from the *a priori* s.d. values of the observations. To make sure that only physically possible VTEC values are obtained, non-negative constraints are introduced. On average, only ~1% of the estimated VTEC values are affected by these constraints, and the impact on the other parameters is negligible. For increased robustness in the estimation, a simple outlier test based on the normalized residuals *υ*/*σ*_*υ*_ is applied. Hobiger *et al.*^34^ stated that a VTEC precision of ~1 TECU can be expected for standard VLBI sessions. In our estimation, we get VTEC *a posteriori* s.e. values of similar size. However, comparisons among the space geodetic techniques suggest that the actual accuracy might be lower^41^.

Besides the parameters of the Earth ionosphere, also the unknowns of the electron density model of the Sun’s corona (*N*_0_ and *β*) and one instrumental delay per station are estimated in a combined least-squares adjustment. Depending on the available observational data, the parameters *N*_0_ and *β* can be strongly correlated. To avoid singularities in our solutions, one of the parameters (usually *β*) is fixed to an *a priori* value.

## Author contributions

B.S. developed the methodology with inputs from all authors, performed the data analysis and wrote the manuscript. All authors reviewed the manuscript.

## Additional information

**How to cite this article:** Soja, B. *et al.* Probing the solar corona with very long baseline interferometry. *Nat. Commun.* 5:4166 doi: 10.1038/ncomms5166 (2014).

## Supplementary Material

Supplementary Movie 1 Coronagraph movie for RD1206. All images recorded by the LASCO C3 coronagraph (ref. 28) during VLBI experiment RD1206, superimposed by the positions of the observed radio sources (white crosses), are merged into this movie. More details about the LASCO C3 images are given in the caption of Figure 3. The three radio sources within LASCO C3's field-of-view are located behind regions of low electron density and turbulence.

Supplementary Movie 2 Coronagraph movie for RD1208. LASCO C3 movie and observed radio sources for session RD1208, similar to Supplementary Movie 1. Both depicted radio sources have lines-of-sight passing through dense regions of the corona. The observations to radio source 1243-072 are affected by the occurrence of a small CME.

Supplementary Movie 3 Coronagraph movie for RD1201. LASCO C3 movie and observed radio sources for session RD1201, similar to Supplementary Movie 1. Near the end of the session, the plasma of a CME reaches the lines-of-sight of radio sources 2008-159 and 1958-179.

Supplementary Movie 4 Coronagraph movie for RD1205. LASCO C3 movie and observed radio sources for session RD1205, similar to Supplementary Movie 1. A CME takes place but the session ends before the plasma can reach the lines-of-sight of the observed radio sources.

## Figures and Tables

**Figure 1:**
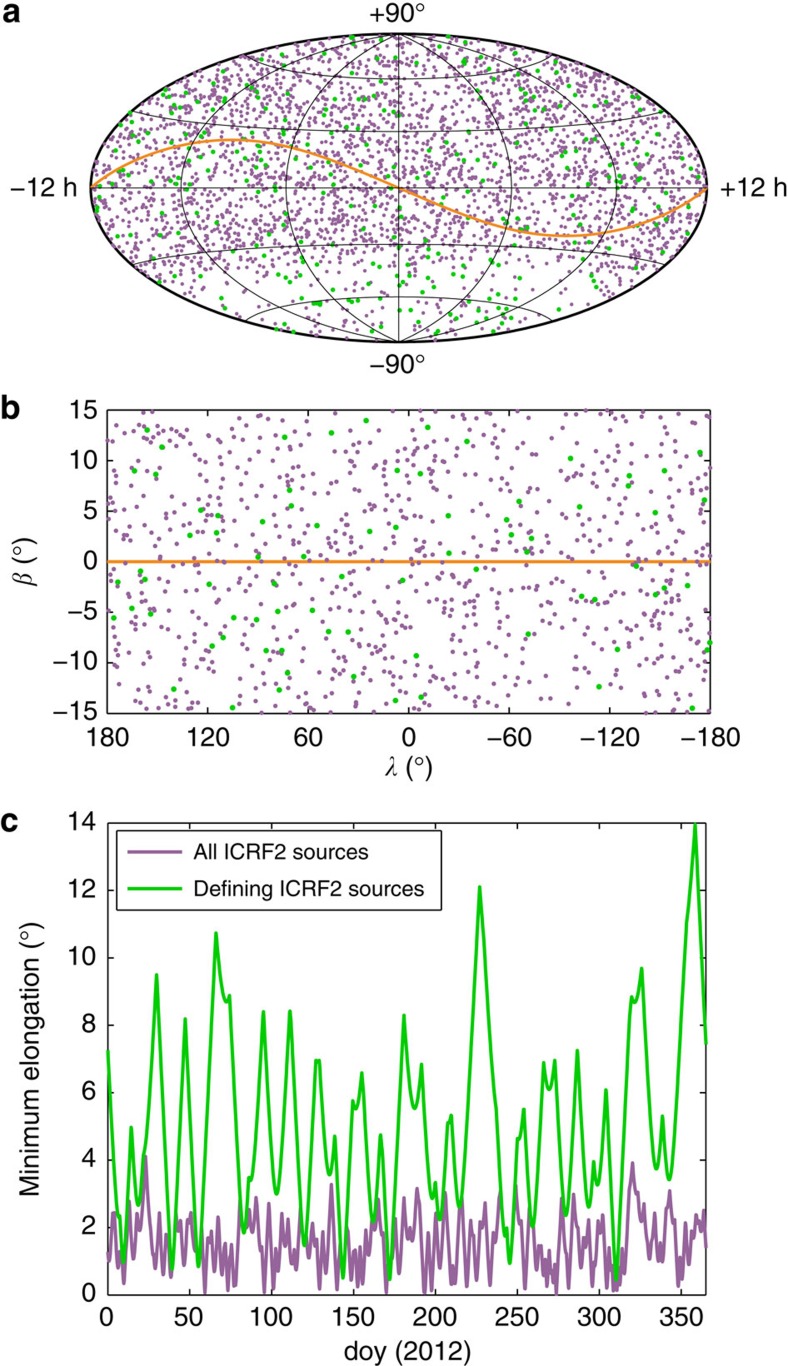
ICRF2 radio sources and minimum elongation angles. Plot **a** shows the defining radio sources (green) and all other radio sources (purple) of the ICRF2 in an equatorial system. The defining radio sources are of high astrometric quality and their positions define the axes directions of the frame. They are preferred for scheduling VLBI experiments. The orange line represents the apparent movement of the Sun as seen from Earth. Plot **b** only includes the 962 radio sources within 15° elongation to the ecliptic, out of which 79 are defining sources. Here, an ecliptic coordinate system is used. The minimum angular distance between the Sun and the ICRF2 radio sources for each day of the year (doy), exemplarily for 2012, is displayed in plot **c**.

**Table 1:** Station networks and observations close to the Sun.

**Session**	**VLBI telescopes**	**Radio sources within 15° (no. of observations)**
RD1106	HhKkMaNyOnTcTsWfWz	1519−273 (9), 1602−115 (5), 1622−253 (13), 1706−174 (6)
RD1107	KkMaNyOnShTsWfWz	1622−253 (24), 1657−261 (2), 1706−174 (3), NRAO530 (30)
RD1201	HhKkNyOnTcTsWfWz	1920−211 (16), 1936−155 (4), 1958−179 (7), 2008−159 (4)
RD1202	HhKkNyOnTcTsWfWz	0019+058 (10), 0055−059 (2), 0111+021 (9), 0119+041 (3), 0119+115 (12), IIIZW2 (3)
RD1203	FtKkMaNyTcTsWz	0342+147 (2), 0406+121 (2), 0440+345 (12), 0446+112 (29), 0506+101 (3), 0515+208 (4)
RD1204	FtMaNyTcTsWz	0515+208 (8), 0528+134 (13), 0536+145 (2), 0544+273 (2), 0600+177 (6), 0611+131 (1)
RD1205	FtHhKkMaNyTsWfWz	0657+172 (65), 0743+277 (3), 0745+241 (18), 0748+126 (98), 0759+183 (2)
RD1206	HhKkNyOnTcTsWfWz	1012+232 (34), 1013+054 (14), 1015+057 (25), 1022+194 (6), 1023+131 (35), 1049+215 (7), 1055+018 (43), 1111+149 (29)
RD1207	HhKkNyOnTcWfWz	1130+009 (11), 1145−071 (1), 1149−084 (19), 1219+044 (18), 1236+077 (20), 1243−072 (15), 3C274 (36)
RD1208	HhKkNyOnTcTsWz	1145−071 (4), 1149−084 (14), 1213−172 (16), 1219+044 (17), 1236+077 (31), 1243−072 (21)
RD1209	HhKkNyOnTcTsWfWz	1519−273 (9), 1602−115 (9), 1622−253 (25), 1657−261 (5), 1706−174 (9)
RD1210	HhKkMaNyOnTsWfWz	1622−253 (19), 1657−261 (12), 1706−174 (15), NRAO530 (34)

The VLBI stations are specified by their two-character IVS codes that can be found at http://ivscc.gsfc.nasa.gov/about/org/components/ns-list.html. For each radio source with elongation angle <15°, the number of successful observations is given. During each session, many other ICRF2 radio sources were observed for a better sky coverage, not included in this table.

**Table 2:** Characteristics of the VLBI experiments.

**Session**	**Date**	**Minimum elongation**	**No. of observations within 15°**	**Total no. of observations**
RD1106	29 Nov 2011	3.9°	33	3,695
RD1107	06 Dec 2011	4.0°	59	4,242
RD1201	24 Jan 2012	4.8°	31	3,482
RD1202	03 Apr 2012	5.8°	39	2,776
RD1203	30 May 2012	10.5°	52	2,099
RD1204	19 Jun 2012	4.4°	32	828
RD1205	10 Jul 2012	6.1°	186	2,953
RD1206	28 Aug 2012	3.9°	193	1,558
RD1207	25 Sep 2012	6.1°	120	1,727
RD1208	02 Oct 2012	3.9°	103	1,918
RD1209	27 Nov 2012	4.2°	57	2,731
RD1210	11 Dec 2012	4.7°	80	3,540

The IVS R&D sessions in 2011 and 2012, which include observations close to the Sun starting at either 17:30 or 18:00 UT on the day given in the second column and lasting 24 h. For each VLBI session, the minimum Sun elongation and the number of successful observations in total as well as closer than 15° are shown.

**Table 3:** Electron density models and statistical measures.

**Session**	**Reduced** ***χ***^**2**^	**ΔAIC**	**Relative likelihood**	***N***_**0**_ **(10**^**12**^ **m**^**−3**^**)**
RD1106	1.1	−5.1	0.08	0.0±0.4
RD1107	1.1	−49.5	0.00	1.7±0.4
RD1201	1.7	−8.4	0.02	3.3±1.3
RD1202	1.2	−0.3	0.85	0.9±0.4
RD1203	1.2	−2.2	0.33	2.2±1.8
RD1204	0.8	−6.4	0.04	1.2±1.0
RD1205	0.8	−4.1	0.13	0.5±0.3
RD1206	0.5	−8.4	0.02	0.3±0.1
RD1207	2.4	0.9	0.65	0.6±0.8
RD1208	2.0	−64.2	0.00	1.5±0.4
RD1209	0.6	−2.8	0.25	0.1±0.3
RD1210	0.6	−16.8	0.00	2.5±0.6
Weighted mean over all R&D sessions				0.57±0.18

For each of the IVS R&D sessions in 2011 and 2012, a least-squares adjustment was performed estimating the parameters of the Sun’s corona, the Earth’s ionosphere and instrumental delays. The goodness of fit is indicated by the reduced *χ*^2^. Another adjustment without estimating the electron density parameter *N*_0_ was performed and for both approaches, the Akaike Information Criterion (AIC) was computed. The difference ΔAIC=AIC_corona_–AIC_without_ is given as well as the relative likelihood exp(ΔAIC/2) of the model excluding the solar corona with respect to the one which takes the corona into account (vice versa for RD1207). Furthermore, the estimated parameters *N*_0_ of the electron density model and their 1*σ* s.e. from the least-squares adjustment (obtained from the *a posteriori* variance-covariance matrix) assuming *β*=2 are listed.

## References

[b1] Aschwanden M. Physics of the Solar Corona Springer (2004).

[b2] Tyler G. L., Brenkle J. P., Komarek T. A. & Zygielbaum A. I. The Viking solar corona experiment. J. Geophys. Res. 82, 4335–4340 (1977).

[b3] Thompson A., Moran J. & Swenson G. Interferometry and Synthesis in Radio Astronomy 2nd edn Wiley (2001).

[b4] Schuh H. & Böhm J. in Sciences of Geodesy II: Innovations and Future Developments (ed. Xu G. 339–376Springer (2013).

[b5] Ho C. & Morabito D. in:DSMS Telecommunications Link Design Handbook, 810-005, Rev. E chapter 106, NASA/DSN (2010).

[b6] Bird M. K. *et al.* The coronal electron density distribution determined from dual-frequency ranging measurements during the 1991 solar conjunction of the ULYSSES spacecraft. Astrophys. J. 426, 373–381 (1994).

[b7] Verma A. K. *et al.* Electron density distribution and solar plasma correction of radio signals using MGS, MEX, and VEX spacecraft navigation data and its application to planetary ephemerides. Astronom. Astrophys. 550, A124 (2013).

[b8] Pätzold M., Bird M. K., Edenhofer P., Asmar S. W. & McElrath T. P. Dual-frequency radio sounding of the solar corona during the 1995 conjunction of the Ulysses spacecraft. Geophys. Res. Lett. 22, 3313–3316 (1995).

[b9] Berman A. L. Electron Density in the Extended Corona—Two Views. Deep Space Network Prog. Rep., 42–41, Jet Propulsion Laboratory, Pasadena, California, USA (1977).

[b10] Esposito P. B., Edenhofer P. & Lueneburg E. Solar corona electron density distribution. J. Geophys. Res. 85, 3414–3418 (1980).

[b11] Hayes A. P., Vourlidas A. & Howard R. A. Deriving the electron density of the solar corona from the inversion of total brightness measurements. Astrophys. J. 548, 1081–1086 (2001).

[b12] Frazin R. A., Vásquez A. M., Kamalabadi F. & Park H. Three-dimensional tomographic analysis of a high-cadence LASCO-C2 polarized brightness sequence. Astrophys. J. 671, L201–L204 (2007).

[b13] Weisberg J. M., Rankin J. M., Payne R. R. & Counselman C. C. Further changes in the distribution of density and radio scattering in the solar corona in 1973. Astrophys. J. 209, 252–258 (1976).

[b14] Shestov S., Urnov A., Kuzin S., Zhitnik I. & Bogachev S. Electron density diagnostics for various plasma structures of the solar corona based on Fe XI-Fe XIII lines in the range 176-207 Å measured in the SPIRIT/CORONAS-F experiment. Astronom. Lett. 35, 45–56 (2009).

[b15] Muhleman D. O., Ekers R. D. & Fomalont E. B. Radio interferometric test of the general relativistic light bending near the sun. Phys. Rev. Lett. 24, 1377–1380 (1970).

[b16] Bird M. *et al.* Coronal radio sounding experiments with the ESA spacecraft MEX, VEX, and Rosetta. *511th WE Heraeus Sem. Bad Honnef, Germany, 31 Jan to 3 Feb 2012* (2012).

[b17] Sovers O. J. Observation Model and Parameter Partials for the JPL VLBI Parameter Estimation Software ‘MODEST’-1991. JPL Publications 83–31 Rev. 4 (1991).

[b18] Schuh H. & Behrend D. VLBI: A fascinating technique for geodesy and astrometry. J. Geodyn. 61, 68–80 (2012).

[b19] Heinkelmann R. in:Geodetic Sciences—Observations, Modelling and Applications ed. Jin S. Ch. 3InTech (2013).

[b20] The Second Realization of the International Celestial Reference Frame by Very Long Baseline Interferometry (eds Fey, A. L., Gordon D., & Jacobs C. S.) IERS Technical Note 35. *Tech. Rep.* (Verlag des Bundesamtes für Kartographie und Geodäsie (2009).

[b21] Soja B., Plank L. & Schuh H. inProc. Journées 2011 Syst. Réf. Spatio-temporels (eds Schuh H., Böhm S., Nilsson T., Capitaine N. 41–44 (Vienna University of Technology, (2012).

[b22] Heinkelmann R. & Schuh H. inProc. IAU Symp. 261 Relativity Fundamental Astronom. Dynam. Ref. Frames Data Anal (eds Klioner S., Seidelmann P. K., Soffel M. 286–290 (Vienna University of Technology, (2009).

[b23] Lambert S. B. & Poncin-Lafitte C. L. Improved determination of γ by VLBI. Astronom. Astrophys. 529, 4 pp (2011).

[b24] Sun J., Pany A., Nilsson T., Böhm J. & Schuh H. inProc. 20th Meeting European VLBI Group Geodes. Astromet (eds Alef W., Bernhart S., Nothnagel A. 44–48 (Max Planck Institute for Radio Astronomy, (2011).

[b25] Akaike H. A new look at the statistical model identification. IEEE Trans. Autom. Control 19, 716–723 (1974).

[b26] Soja B., Heinkelmann R. & Schuh H. Solar corona electron densities from VLBI and GIM data. Int. Assoc. Geodes. Symp. 143, Springer (2014).

[b27] Pätzold M. *et al.* Coronal density structures and CMEs: superior solar conjunctions of Mars Express, Venus Express, and Rosetta: 2004, 2006, and 2008. Solar Phys. 279, 127–152 (2012).

[b28] Brueckner G. E. *et al.* The large angle spectroscopic coronagraph (LASCO). Solar Phys. 162, 357–402 (1995).

[b29] Ma C. inInt. VLBI Service Geodes. Astromet. 2010 Gen. Meeting Proc (eds Behrend D., Baver K. 273–279 (NASA, Goddard Space Flight Center (2010).

[b30] Petrachenko B. *et al.* Design aspects of the VLBI2010 system. ftp://ivscc.gsfc.nasa.gov/pub/misc/V2C/TM-2009-214180.pdf (NASA, 2009).

[b31] Spangler S. R. *et al.* Very long baseline interferometer measurements of turbulence in the inner solar wind. Astron. Astrophys. 384, 654–665 (2002).

[b32] Sovers O. J., Fanselow J. L. & Jacobs C. S. Astrometry and geodesy with radio interferometry: experiments, models, results. Rev. Mod. Phys. 70, 1393–1454 (1998).

[b33] Ma C. *Very Long Baseline Interferometry Applied to Polar Motion, Relativity and Geodesy*. PhD thesis, Univ. Maryland (1978).

[b34] Hobiger T., Kondo T. & Schuh H. Very long baseline interferometry as a tool to probe the ionosphere. Radio Sci. 41, RS1006 (2006).

[b35] Hawarey M., Hobiger T. & Schuh H. Effects of the 2nd order ionospheric terms on VLBI measurements. Geophys. Res. Lett. 32, L11304 (2005).

[b36] Hobiger T. *VLBI as a Tool to Probe the Ionosphere*. PhD thesis, Vienna Univ. Technology (2006).

[b37] Sakurai T., Spangler S. R. & Armstrong J. W. Very long baseline interferometer measurements of plasma turbulence in the solar wind. J. Geophys. Res. 97, 17141–17151 (1992).

[b38] Schaer S. *Mapping and Predicting the Earth’s Ionosphere Using the Global Positioning System*. PhD thesis, Astronomical Institute, Univ Berne (1999).

[b39] Kondo T. Application of VLBI data to measurements of ionospheric total electron content. J. Commun. Res. Lab. 38, 613–622 (1991).

[b40] Dettmering D., Schmidt M., Heinkelmann R. & Seitz M. Combination of different space-geodetic observations for regional ionosphere modeling. J. Geod. 85, 989–998 (2011).

[b41] Dettmering D., Heinkelmann R. & Schmidt M. Systematic differences between VTEC obtained by different space-geodetic techniques during CONT08. J. Geod. 85, 443–451 (2011).

