# Supplementary figures and images for: Probing the solar corona with very long baseline interferometry
Source: Nat Commun. 2014 Jun 20;5:4166. doi: 10.1038/ncomms5166 (PMC4279185; doi:10.1038/ncomms5166)

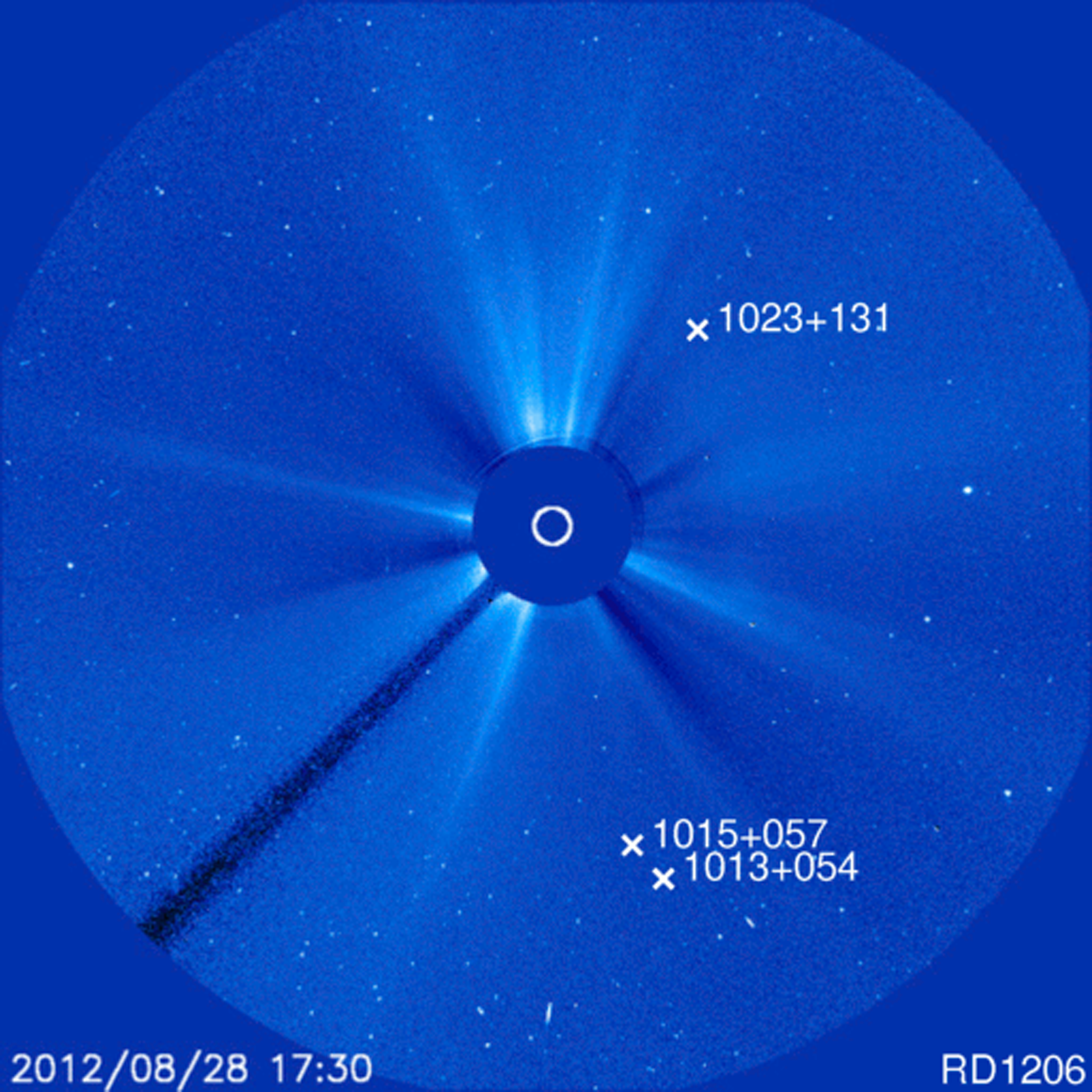

Supplement: Supplementary Movie 1 — Coronagraph movie for RD1206. All images recorded by the LASCO C3 coronagraph (ref. 28) during VLBI experiment RD1206, superimposed by the positions of the observed radio sources (white crosses), are merged into this movie. More details about the LASCO C3 images are given in the caption of Figure 3. The three radio sources within LASCO C3's field-of-view are located behind regions of low electron density and turbulence. [file ncomms5166-s1.tif]

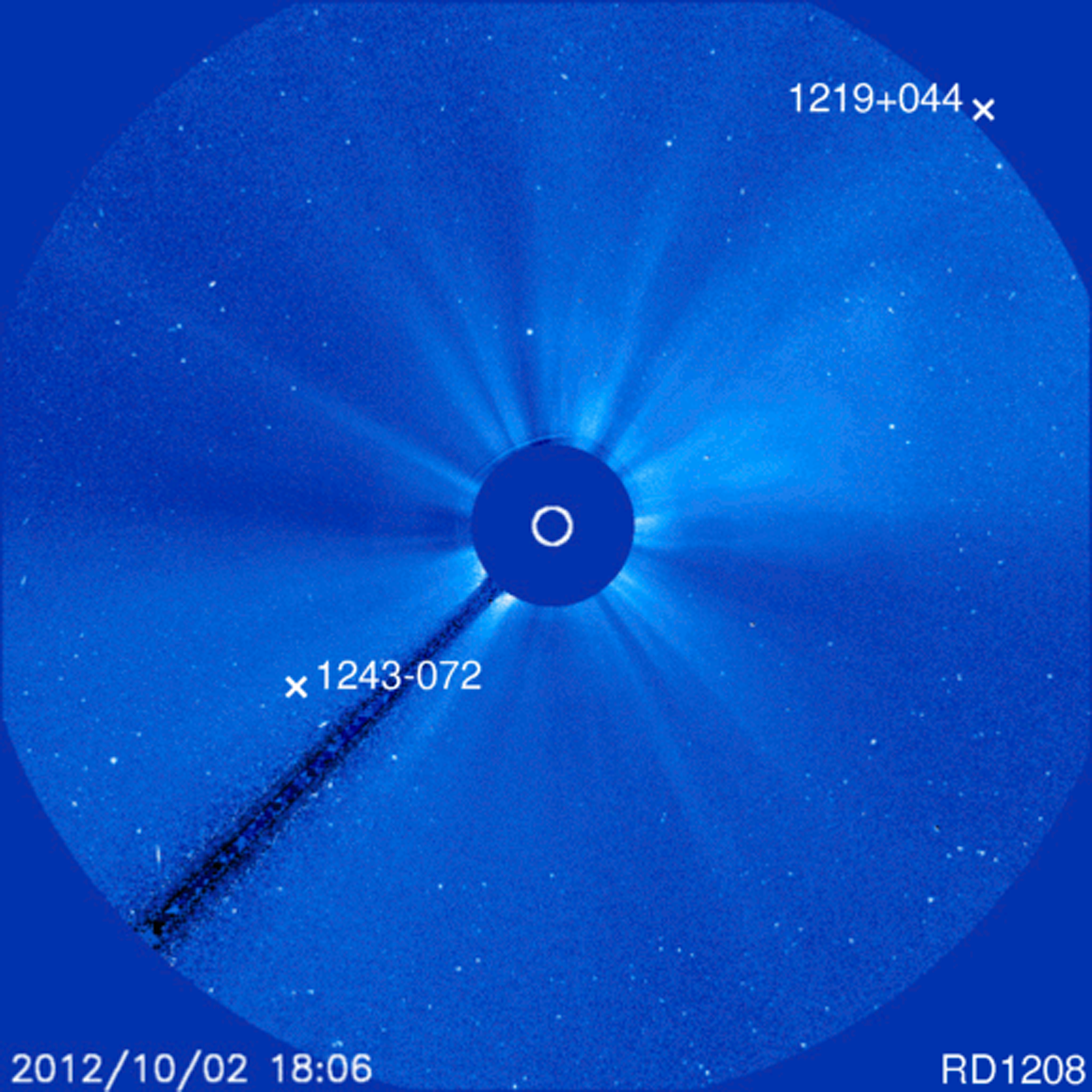

Supplement: Supplementary Movie 2 — Coronagraph movie for RD1208. LASCO C3 movie and observed radio sources for session RD1208, similar to Supplementary Movie 1. Both depicted radio sources have lines-of-sight passing through dense regions of the corona. The observations to radio source 1243-072 are affected by the occurrence of a small CME. [file ncomms5166-s2.tif]

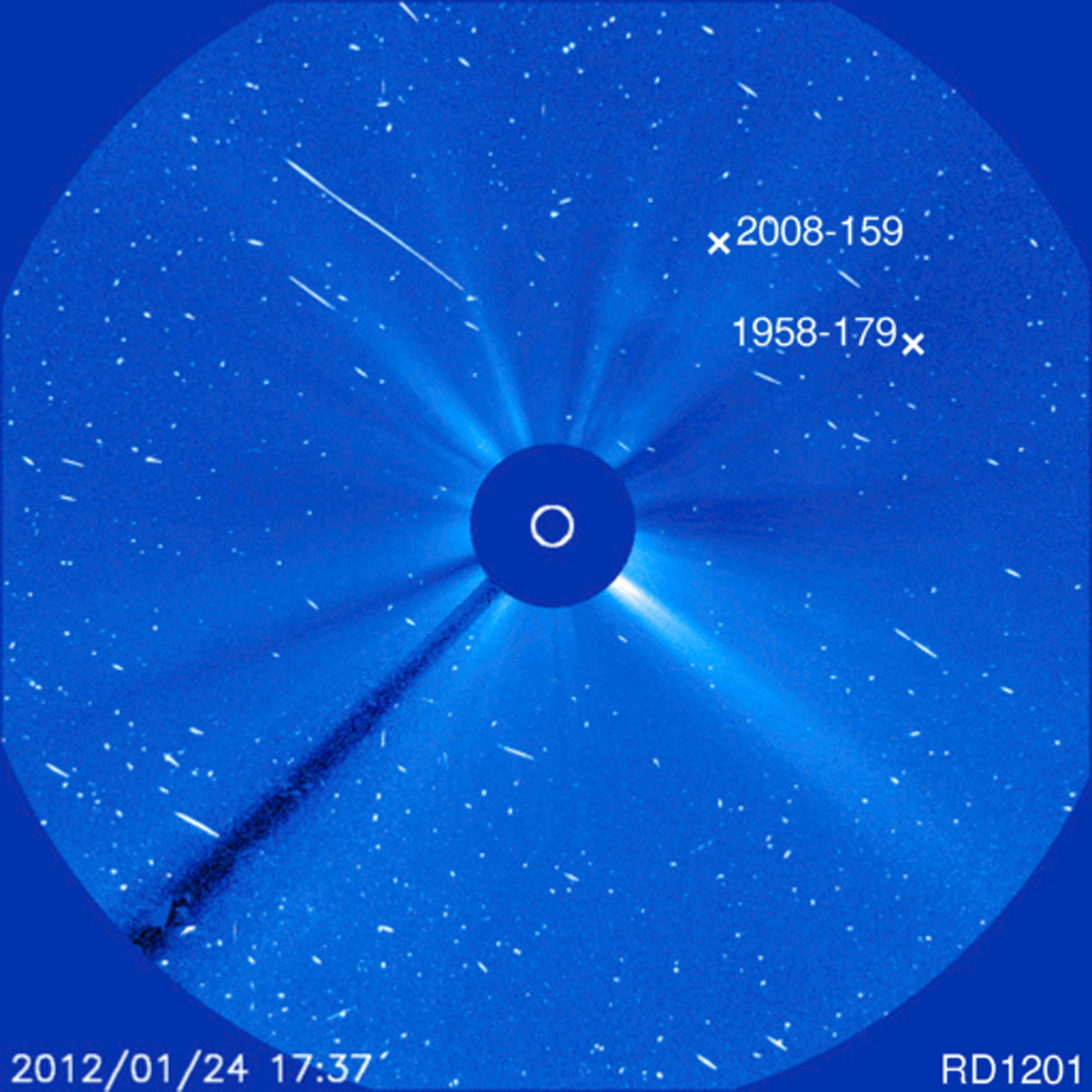

Supplement: Supplementary Movie 3 — Coronagraph movie for RD1201. LASCO C3 movie and observed radio sources for session RD1201, similar to Supplementary Movie 1. Near the end of the session, the plasma of a CME reaches the lines-of-sight of radio sources 2008-159 and 1958-179. [file ncomms5166-s3.tif]

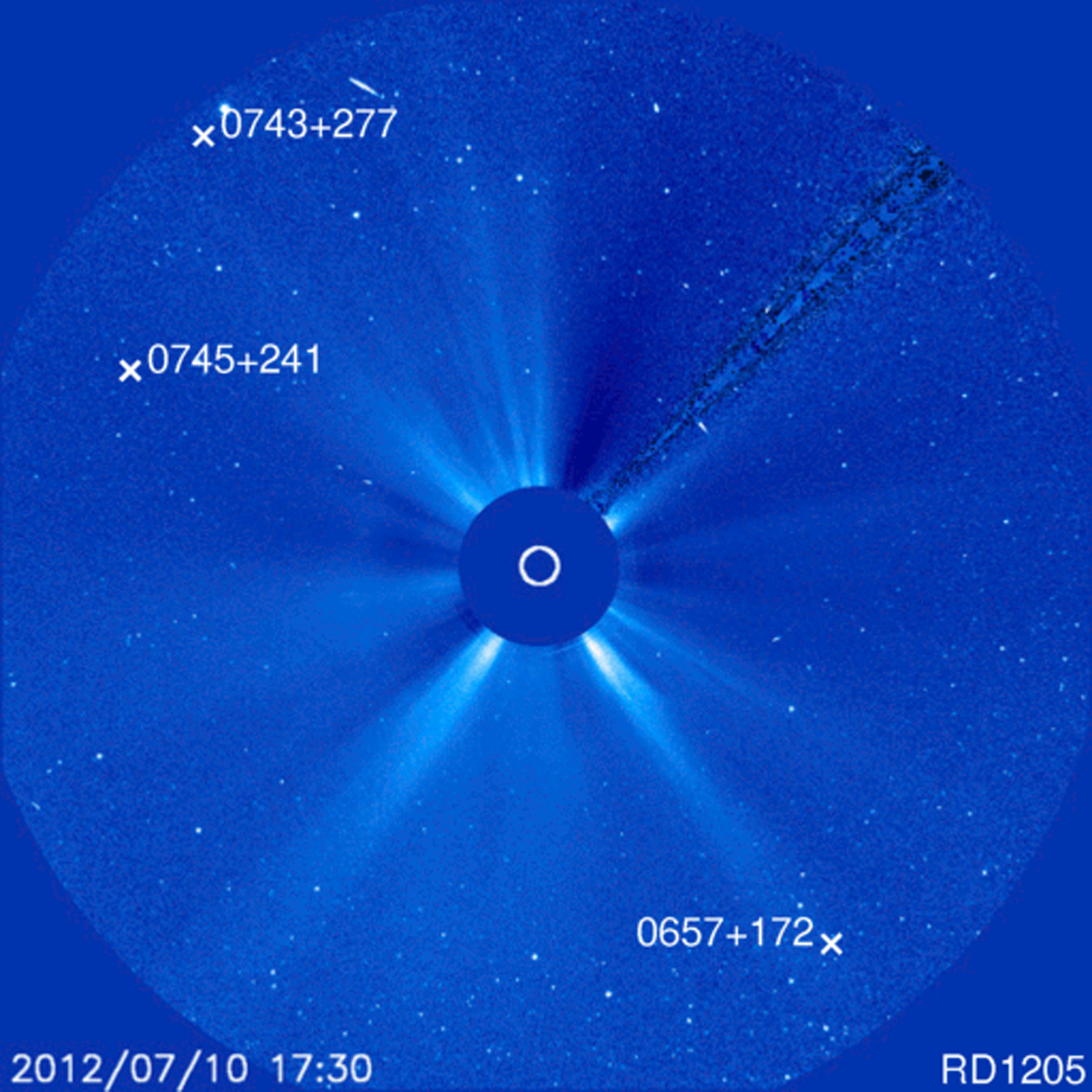

Supplement: Supplementary Movie 4 — Coronagraph movie for RD1205. LASCO C3 movie and observed radio sources for session RD1205, similar to Supplementary Movie 1. A CME takes place but the session ends before the plasma can reach the lines-of-sight of the observed radio sources. [file ncomms5166-s4.tif]
